# COVID-19 Vaccine Platforms: Challenges and Safety Contemplations

**DOI:** 10.3390/vaccines9101196

**Published:** 2021-10-18

**Authors:** Hadeel T. Al-Jighefee, Hoda Najjar, Muna Nizar Ahmed, Abeer Qush, Sara Awwad, Layla Kamareddine

**Affiliations:** 1 Department of Biomedical Science, College of Health Sciences, QU Health, Qatar University, Doha P.O. Box 2713, Qatar; ha1510267@student.qu.edu.qa (H.T.A.-J.); hn1517144@student.qu.edu.qa (H.N.); ma1507488@student.qu.edu.qa (M.N.A.); 200050311@student.qu.edu.qa (A.Q.); sa1900788@student.qu.edu.qa (S.A.); 2 Biomedical Research Center, Qatar University, Doha P.O. Box 2713, Qatar; 3 Biomedical and Pharmaceutical Research Unit, QU Health, Qatar University, Doha P.O. Box 2713, Qatar

**Keywords:** SARS-CoV-2, COVID-19, vaccine platforms, challenges, safety, strengths, limitations

## Abstract

The severe acute respiratory syndrome coronavirus 2 (SARS-CoV-2) has become a pandemic as of March 2020, creating a global crisis and claiming millions of lives. To halt the pandemic and alleviate its impact on society, economy, and public health, the development of vaccines and antiviral agents against SARS-CoV-2 was a dire need. To date, various platforms have been utilized for SARS-CoV-2 vaccine development, and over 200 vaccine candidates have been produced, many of which have obtained the United States Food and Drug Administration (FDA) approval for emergency use. Despite this successful development and licensure, concerns regarding the safety and efficacy of these vaccines have arisen, given the unprecedented speed of vaccine development and the newly emerging SARS-CoV-2 strains and variants. In this review, we summarize the different platforms used for Coronavirus Disease 2019 (COVID-19) vaccine development, discuss their strengths and limitations, and highlight the major safety concerns and potential risks associated with each vaccine type.

## 1. Introduction

**Figure 1:**
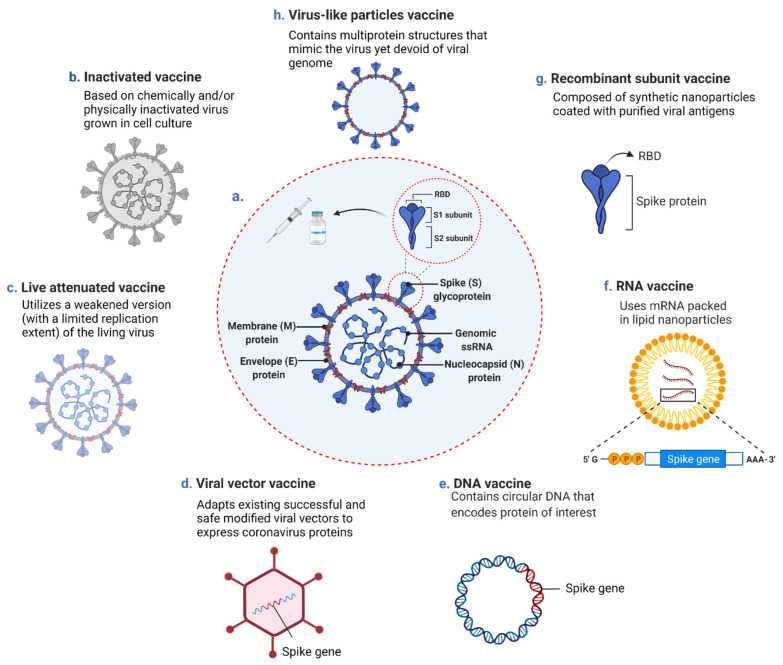
SARS-CoV-2 structure and contemporary COVID-19 vaccine platforms. (**a**) Schematic diagram of SARS-CoV-2 structure including the single-stranded RNA (ssRNA) genome and the four structural proteins: spike protein (S), envelope protein (E), membrane protein (M), and nucleocaspid protein (N). Diverse vaccine platforms including (**b**) inactivated vaccine (**c**) live attenuated vaccine (**d**) viral vector vaccine (**e**) DNA vaccine (**f**) RNA vaccine (**g**) recombinant subunit vaccine (**h**) virus-like particles vaccine. mRNA: messenger RNA, RBD: receptor-binding domain. The diagram was created with BioRender.com.

The coronavirus disease 2019 (COVID-19), caused by the novel severe acute respiratory syndrome coronavirus 2 (SARS-CoV-2), first reported in late 2019 in Wuhan, the capital of Hubei province in Central China, has become a global pandemic with devastating effects worldwide [1]. Since then, and until 29 June 2021, this newly emerging disease caused by the enveloped SARS-CoV-2 virus, which belongs to the Coronaviridae family and the lineage B of the betacoronavirus (β-CoV) genera, has brought over 181 million confirmed cases and claimed the lives of about 4 million people worldwide [1]. SARS-CoV-2 has a positive-sense, single-stranded genome that encodes a large non-structural polyprotein (ORF1a/b) proteolytically cleaved to generate proteins, four of which are structural proteins including spike (S), envelope (E), membrane (M), and nucleocapsid (N) (Figure 1a) [2,3]. Among these proteins, the S surface glycoprotein plays a critical role in receptor recognition and attachment to host cells [4]. The S protein also induces T-cell responses and is the main target of highly potent neutralizing antibodies (nAbs) against the virus, presenting it as the major antigenic pick out for vaccine design [5]. The structure of SARS-CoV-2 is similar to other β-CoVs, including the severe acute respiratory syndrome coronavirus (SARS-CoV) and the Middle Eastern respiratory syndrome-related coronavirus (MERS-CoV), the causative agents of SARS and MERS, two previously reported viral pneumonia disease outbreaks, respectively [6]. Compared to SARS-CoV and MERS-CoV; however, SARS-CoV-2 has higher infectivity and transmissibility due to its high-affinity binding to the host cell receptors and high viral shedding levels during the early stage of infection, contributing to the vastly infectious nature of asymptomatic and mildly symptomatic patients [7,8,9]. As initial measures to control the disease spread, the COVID-19 pandemic was primarily withstood through social distancing, hygiene measures, and repurposed drugs [10]. Some countries’ implemented measures were relatively emollient and particularly designated to control the disease by achieving herd immunity following natural infection [11,12].

**Figure 2:**
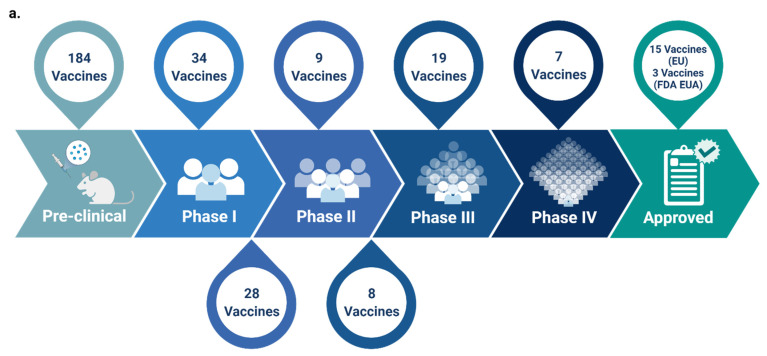

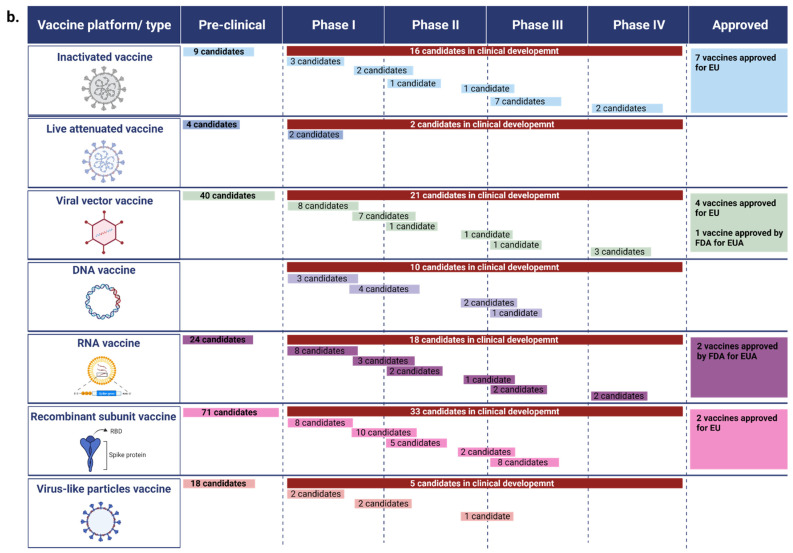
Phases of COVID-19 vaccine development. (**a**) The total number of currently available COVID-19 vaccine candidates in the pre-clinical and clinical phases of development. (**b**) Number of developed COVID-19 vaccine candidates per vaccine platform. Data was retrieved from the World Health Organization (WHO) vaccine tracker and landscape website [26] on 29 June 2021. N.B. The reported numbers are subject to change with time given the current efforts and pace of COVID-19 vaccines development. EU: emergency use, FDA: food and drug administration, EUA: emergency use authorization. The figure and tablewere created with BioRender.com.

Therefore, despite the taken measures and as a consequence of not implementing immediate lockdown, the COVID-19 death toll increased [13,14]. This necessitated the development of an effective and safe vaccine as an imperative solution to control the pandemic and prevent future outbreaks [13,15]. As such, and since the release of the SARS-CoV-2 genome sequence in January 2020, all efforts have been directed towards COVID-19 vaccines development [16,17]. The hope and hype placed on vaccines to prevail over the disease stand up from the success of previously developed vaccines to control other infectious diseases [13]. The route for vaccine development; however, was not always paved, and several historical attempts of vaccines production were doomed with defeats [18]. Until today, and despite all the knowledge and technology at one’s disposal, scientists are still unable to conclude the safest and most effective vaccine platform [18]. Back in time, particularly following the outbreak of SARS-CoV in 2002, vaccines against the emerging virus were also developed, a few of which reached phase I clinical trials; yet, did not achieve the final stages and obtain the United States Food and Drug Administration (FDA) approval as the virus was eradicated from the human population in 2004 [16,19,20,21]. Similarly, several vaccines against MERS-CoV were under development, none of which have obtained FDA approved thus far [21]. Within the same notion, and in relay for safe and effective COVID-19 vaccine production, censorious steps are currently followed in all phases of COVID-19 vaccine development, including manufacturing, dispersal, and vaccination [22]. For the time being, many of the newly developed COVID-19 vaccines are undergoing clinical evaluation and have reached phase III of clinical trials. A few of which have been approved for emergency use [13] (Figure 2a, Table 1), with the research and discovery phase being skipped [21,23]. Several approaches, including traditional platforms (inactivated and live attenuated virus vaccines), and newly established ones (replicating and non-replicating viral vector vaccines, nucleic acid (DNA and RNA) vaccines, recombinant subunit vaccines, and peptide-based/virus-like particles vaccines), have been adopted for COVID-19 vaccine development (Figure 1b–h) [16,24,25]. As of 29 June 2021, and according to the World Health Organization (WHO), out of the 293 total COVID-19 vaccine candidates, 105 are currently in the clinical phase of development and 184 are still in the pre-clinical phase (Figure 2a) [26].Presently, and besides the FDA consideration of the possibility of booster vaccine shots, several standpoints are now advocating the notion that “hybrid immunity” and “the mix and match of different vaccines strategy” could provide an even stronger immune boost, presenting such approaches, if supported by data, as plausible pandemic game-changers. In this review, we detail the different COVID-19 vaccine platforms and highlight their strengths, limitations, and major risks and safety concerns associated with each type, particularly those relevant to the fast-track pace taken for their production. We also summarize all candidate COVID-19 vaccines currently in the clinical phase of development and categorize them according to the platform used for their development.

## 2. Contemporary COVID-19 Vaccine Platforms and Allied Safety and Efficacy Concerns

### 2.1. Inactivated Vaccine

Purified inactivated viruses have been widely used for over a century in vaccine development against various emerging infectious diseases, including influenza, polio, rabies, and hepatitis A [27,28,29,30,31]. Today, inactivated vaccines are typically produced by propagating the virus in cell culture systems, followed by purification, concentration, and chemical and/or physical inactivation to demolish infectivity while retaining immunogenicity (Figure 1b) [32,33]. This type of vaccine is notably featured by its highly efficient proliferation and genetic stability [34]; yet, limited by the viral yield in a cell culture setting, the requirement of a biosafety level 3 facility, and the short duration of the elicited immune response, possibly making the vaccines less effective in preventing viral entry [33,35]. Up to date, 16 inactivated SARS-CoV-2 vaccines have been developed and are currently in clinical trial phases (Figure 2b) [26]. One of which, for example, is the Sinovac’s CoronaVac vaccine candidate which has demonstrated sufficient safety and efficacy in phase III of clinical trials in Brazil, Turkey, and Indonesia and is currently in phase IV of clinical trials (Table 1) [26,34,36,37,38,39]. Another is the BBIBP-CorV vaccine candidate, which showed adequate humoral immune responses in adults aged 18 years and above and currently stands in phase IV of clinical trials (Table 1) [39,40,41]. Both vaccines have been listed by the WHO for COVID-19 Emergency Use (EUL) and are presently being adopted by several countries worldwide. Despite these promising data, concerns of using inactivated virus vaccines platforms against COVID-19 still reside, some of which relate to the difficulty of confirming a complete virus inactivation status, a risk that could translate into a scenario similar to the 1955 Cutter incident where children receiving the polio vaccine were infected with the inactivated poliovirus [33,42]. Into the bargain, although several developed inactivated SARS-CoV vaccines have been reported to induce nAbs, vaccinated animals still display significant disease upon challenge, which could explain why no vaccines are currently licensed for SARS-CoV [43]. Further, previous studies on animal models have shown that immunizations with inactivated SARS-CoV and MERS-CoV vaccines are associated with hypersensitive-type lung pathology post-challenges with the infectious virus [32,44,45,46]. Similarly, respiratory syncytial virus (RSV) formalin-inactivated vaccine has been reported to cause enhanced pulmonary disease after live RSV infection [47,48]. In addition, it was suggested that treating the vaccine with formalin could have altered the epitopes, inducing functional antibodies, causing the immune system to produce antibodies against non-protective epitopes [33,49]. It is worth noting here that none of these concerns and/or complications of using inactivated virus vaccines have been thus far reported from the use of recently developed COVID-19 inactivated vaccines.

### 2.2. Live Attenuated Vaccine

Live attenuated vaccines, which embody a weakened version of the live virus with reduced virulence, are considered one of the oldest and most effective immunization approaches to elicit life-long immune responses (Figure 1c) [32,50]. A remarkable advantage of such a vaccine type is its relatively low production and delivery costs, given that the attenuated virus can replicate and propagate within the host. As such, a relatively small dose of the virus can be enough to induce immunity [51]. Moreover, live attenuated vaccines can be given intranasally, allowing the attenuated virus to replicate in the mucosal tissue of the upper respiratory tract, a major portal for coronaviruses entry into the host [52]. For the time being, only six SARS-CoV-2 live attenuated virus vaccines have been developed, four of which are in the pre-clinical phase, and two are in phase I of clinical trials (Figure 2b, Table 1) [26]. Both COVI-VAC and MV-014-212 vaccines are attenuated via codon pair deoptimization, a strategy that involves synthetic recoding of the viral genome by amending the positions of synonymous codons, thereby raising the number of suboptimal codon pairs and cytosine phosphoguanine (CpG) dinucleotides in the recoded genome [25,53,54,55]. In parallel to live attenuated SARS-CoV-2 vaccine studies, ongoing studies on other live attenuated virus vaccines such as the RSV vaccine have shown success in using the codon pair deoptimization strategy in vaccine production evidenced by the robust humoral and cellular immune responses triggered in non-human primates [56].

Despite the aforementioned advantages and the pulled off accomplishments of using live attenuated virus vaccine in combating different infectious diseases, the overt risk of using such a type of vaccine still resides in the use of a live replicating virus, which can revert under any condition to its pathologic phenotype, causing disease after vaccination, especially in immunocompromised individuals [57,58]. Although this anticipated scenario is considered relatively rare, the degree of unpredictability regarding the virus stability and the arising safety considerations after that should never be ruled out [59]. Further, live attenuated vaccines could result in viral shedding into the environment, posing a potential risk to the unvaccinated community [60]. It also goes without saying that these highlighted disadvantages are acquainted with time consumption and technical difficulties associated with the virus modification approaches if such a vaccine platform is to be implemented [16].

### 2.3. Viral Vector Vaccine

Viral vector vaccines, in both replicating and non-replicating forms, utilize modified viruses such as adenoviruses or poxviruses as the vector to deliver the genetic material coding for a viral antigen of interest into the host cell (Figure 1d) [57,61]. In self-replicating (replication-competent) viral vector-based vaccines, and through the host cell machinery used by the virus vector, new viral particles are produced in infected cells, which then infect other new cells, resulting in additional vaccine antigen production [62]. On the contrary, non-replicating (replication-incompetent or deficient) viral vector-based vaccines cannot produce new viral particles, and the host cell machinery is used to produce the vaccine antigens, after which the viral vector gets cleared [61,62]. Both viral vector vaccine forms do not cause infection from neither the loaded virus nor the viral vector as the delivered genetic material does not become integrated into the host genome [61,63]. Typically, the advantage of this type of vaccine lies in promoting the expression of viral antigens within infected host cells for efficient major histocompatibility complex (MHC) class I and class II presentation [61]. Moreover, viral vectors are characterized by their high gene transduction efficiency, high specificity of genes delivered to target cells, and the immune response they elicit with increased cellular response [64]. Further, although viral vector vaccines are generally considered less robust than traditional vaccine types, the fact that they persist as genetic material in the host, directly infect antigen-presenting cells, and possess a strong inherent adjuvant activity triggering innate and adaptive immune responses and generating high titers of nAbs, could suffice a single vaccine dose for adequate immunization as in the case of the vesicular-stomatitis virus -(VSV)-based Ervebo vaccine against Ebola virus [62,63,65]. In COVID-19 vector-based vaccine production, replicating and non-replicating vectors have been utilized to deliver genes encoding for either the SARS-CoV-2 S glycoprotein or the receptor-binding domain (RBD) [16,26]. Thus far, vaccinia and adenovirus are the predominantly used virus vectors for vectored vaccines development [64]. The adenovirus, for example, has been previously utilized in developing SARS-CoV vaccines expressing the S and N proteins [32,43,66]. Currently, it is also being used for developing COVID-19 vector-based vaccines. Up to date, 4 replicating and 17 non-replicating COVID-19 vector-based vaccines have been developed, of which 2 have reached phase III clinical trials, and 3 are currently in phase IV (Table 1, Figure 2b) [26]. All five vaccines are adenovirus-based non-replicating vaccines containing the gene encoding for SARS-CoV-2 S glycoprotein [67,68,69,70]. Among these vaccines, Janssen’s (Ad26.COV2.S) vaccine has recently received the FDA EUA for use in in 18 years old and elder individuals after showing good efficacy data in phase III of clinical trials [71]. Although the Ad26.COV2.S vaccine showed around 65–66% efficacy in moderate to severe/critical and around 76–83% in severe/critical COVID-19 patients, its efficacy dropped to 52 and 64% against the Beta (B.1.351) variant in moderate to severe/critical disease conditions, respectively [69] (Table 1). Low efficacy data were also reported for AstraZeneca vaccine against the Beta variant, with an efficiency of 10.4% only reported in South Africa and 48% in Canada [72,73], contrarily to the 70.4% retained efficacy against the Alpha (B.1.1.7) variant as reported in a study conducted in the UK [74]. The other three viral vector vaccines at stages II/III–IV of clinical development are CanSino’s adenovirus type-5 (Ad5) vectored vaccine, Gamaleya Research Institute’s Gam-COVID-Vac vaccine, and ReiThera’s GRAd-COV2 (Table 1). Although clinical trials have revealed that these vaccines are tolerable and immunogenic, age and the presence of high pre-existing anti-adenovirus immunity were shown to partly diminish vaccination-induced specific antibody and T-cell responses [68]. To overcome pre-existing immunity to the adenovirus in vaccinated individuals, a plausible approach could be using a heterologous recombinant vector as in the Gam-COVID-Vac (Sputnik V) vaccine, the only heterologous COVID-19 vaccine that uses both adenovirus 26 (Ad26) and adenovirus 5 (Ad5) as vectors to express the SARS-CoV-2 S protein [70,75]. Of note, the general principle of prime-boost with two distinct vectors was not exclusively used in recent COVID-19 vaccine platforms but has been largely implemented experimentally and was also previously used in developing the GamEvac-Combi Ebola virus vaccine [76].

### 2.4. Nucleic Acid (DNA and RNA)-Based Vaccine

In nucleic acid-based vaccines, only the genetic material (DNA or RNA), but not the recombinant/live virus, is taken up by host cells and translated into the protein to elicit an immune response (Figure 1e,f) [77]. Although various messenger RNA (mRNA) vaccines, including those against influenza, Zika, and rabies viruses, have been thus far developed, this vaccine development platform is still considered relatively new [78]. The pronounced advantage of some types of nucleic acid vaccines generally lies in the large-scale production pace and cost [16]. DNA vaccines, for example, are based on the use of highly stable plasmid DNA that can be easily propagated at a large scale in bacteria, as the plasmid DNA typically encloses mammalian expression promoters and the gene encoding the protein of interest [16]. On the other hand, presenting mRNA vaccines as promising alternatives for conventional vaccines mainly lies in the ability to produce the vaccine completely in vivo, along with their high potency, cost-effectiveness, rapid development, and safe delivery [16,78,79]. Currently, lipid nanoparticles (LNPs) are among the most commonly used in vivo RNA delivery vectors, protecting the mRNA from enzymatic degradation and facilitating endocytosis and endosomal escape [80]. Contrarily to the highlighted recognition of mRNA vaccines, the physiochemical properties of the mRNA that may impact its cellular and organ dispersal, the questioned safety and efficacy of mRNA vaccine use in humans, them being unlikely to induce strong mucosal immunity due to their intramuscular administration, and the uncertainty from what could arise with large-scale production, storage, and stability are among the alarming concerns tailored to mRNA vaccines production [16,57,80]. Likewise, potential disadvantages also relate to DNA vaccines, particularly those relevant to their low immunogenicity and to the need of DNA molecules to traverse the nuclear membrane to be transcribed, necessitating complicated delivery systems such as electroporators for better efficiency [16,57]. In addition, introducing mutation and dysregulated gene expression by the plausible stable integration of transfected DNA into the somatic or germline host cells genome is another arising concern [81] though unconventional as per relevant follow-up studies [82,83,84,85]. Up to date, 28 nucleic acids (10 DNA and 18 mRNA)-based COVID-19 vaccines have been developed and are currently in the clinical stages, and 24 mRNA vaccines are in the pre-clinical stage (Figure 2b, Table 1) [26]. Two mRNA-based vaccines, developed by Pfizer/BioNTech and Moderna, are currently in phase IV clinical trials and have received the FDA EUA for protection against COVID-19 [26,86,87]. Preliminary results showed astoundingly 94–95% efficacy for both vaccines [88,89]. Though promising, a major concern relevant to mRNA vaccines resides in their rapid pace of development and the uncertainty of potential long-term adverse effects associated with them, particularly because these are the first approved mRNA vaccines with no other FDA-approved mRNA vaccines to date [90]. Another concern is the efficacy of these vaccines against the newly emerging SARS-CoV-2 variants with mutations in the S protein, the main target in COVID-19 vaccines development [91]. As of yet, Pfizer/BioNTech COVID-19 vaccine was reported to protect against four variants of concern (VOCs), including Alpha, Beta, Gamma, and Delta (Table 1) [91,92,93,94]. Interestingly, a recent study by Zakhartchouk et al. reported that combining DNA vaccine and whole killed virus vaccines augments immune responses to SARS-CoV [95], a propitious tactic worth considering in ongoing COVID-19 vaccine development approaches [95].

### 2.5. Protein Subunit and Virus-Like Particles Vaccine

As compared to the whole-pathogen vaccine platform, a protein subunit vaccine is composed of in vitro harvested and highly purified viral protein antigens carefully chosen for their ability to elicit an immune response (Figure 1g) [96]. Being incapable of causing disease, the protein subunit vaccine platform is considered safer than the whole-virus (live attenuated and inactivated) platforms [97]. Not displaying the full antigenic complexity of the virus and enclosing small antigens deficient of pathogen-associated molecular patterns (PAMPs); however, it may promote skewed immune responses, bringing the immunogenicity potential and protective efficacy of protein subunit vaccines into question [57,97]. Subunit vaccine design and production could be also costly and might necessitate specific adjuvants to boost the immune response [98], in addition to the potential occurrence of antigen denaturation, which could lead to non-specific binding [99]. Examples of developed subunit vaccines include the recombinant RBD subunit vaccine, which was reported to elicit partial protective immunity in rhesus macaques against MERS-CoV challenge [100], and S protein-based subunit vaccines against SARS-CoV infection with potency to induce nAbs and protect against SARS-CoV intranasal infection in mice [32,101]. Up to date, 33 COVID-19 protein subunit vaccines based on the S protein or the RBD have been developed and are in the clinical stages. Of which, 10 vaccines, including Novavax’s (NVX-CoV2373) are in phase III [26,102]. Recent reports showed that a two-dose regimen of the NVX-CoV2373 vaccine exhibited 89.7% efficacy against SARS-CoV-2 infection, with high efficacy against the Alpha, Beta, and other VOCs [102,103] (Table 1). Virus-like particles (VLPs) vaccine is another type of protein-based vaccine composed of proteins from the viral capsid only with no viral genetic material (Figure 1h) [57,104]. In addition to being safe, VLPs elicit potent immune responses due to their repetitive structures [104]. VLP vaccines against many viruses, including Hepatitis B virus, Human papillomaviruses, and Influenza A virus, do exist [104,105,106,107]. Likewise, VLP vaccines against MERS-CoV and SARS-CoV infection have been also developed, with eosinophilic pulmonary immunopathology detected after viral challenge in some cases [21,46,108]. For the COVID-19 status quo particularly, five VLPs vaccines in different phases of clinical trials are thus far available (Figure 2b, Table 1) [26].

**Table 1:** SARS-CoV-2 Vaccine Candidates in Clinical Development Stages.

Platform/Vaccine Type	No.	Vaccine Name	Number of Doses (Dosage)	Dosing Schedule	Route of Administration	Developer/Manufacturer	Construct and/or Targeted SARS-CoV-2 Protein	Current Stage of Clinical Trial (Recruitment Status)	Efficacy *	Current Approvals/Authorizations	Reference
Inactivated virus	1	CoronaVac	2 doses (3 μg)	Day 0 + 14	IM	Sinovac Research and Development Co., Ltd.	Whole inactivated SARS-CoV-2 with aluminum hydroxide adjuvant	Phase IV (Not yet recruiting)	**Efficacy from clinical trials:** **Brazil**: 50.7% against symptomatic disease ≥14 d after 2 doses. **Turkey**: 83.5% against symptomatic disease ≥14 d after 2 doses. **Indonesia**: 65.3% against symptomatic disease ≥14 d after 2 doses. **Efficacy/effectiveness against variants:** **Chile** (predominant circulation of P.1 and B.1.1.7.): 67% against symptomatic disease ≥28 d after 2 doses. **Brazil** (predominant circulation of P.2 and P.1 lineages): 50.7% and 36.8% against symptomatic disease ≥14 d after 2 doses, respectively.	WHO EUL Approved in 37 countries ^1^	[26,36,37,38,109,110,111,112]
Inactivated virus	2	BBIBP-CorV	2 doses (4 μg)	Day 0 + 21	IM	Sinopharm + China National Biotec Group Co + Beijing Institute of Biological Products	Whole inactivated SARS-CoV-2	Phase IV (Recruiting)	**Efficacy from clinical trials in UAE, Bahrain, Egypt, and Jordan:** 78.1% against symptomatic disease ≥14 d after 2 doses, and 79% against hospitalization.	WHO EUL Approved in 56 countries ^2^	[26,34,39,41,110,113]
Inactivated virus	3	Inactivated SARS-CoV-2 vaccine (Vero cell)	2–3 doses (5 μg)	Day 0 + 21 + 42 or 111 or 171	IM	Sinopharm + China National Biotec Group Co + Wuhan Institute of Biological Products	Whole inactivated SARS-CoV-2 with aluminum hydroxide adjuvant	Phase III (Completed)	**Efficacy from clinical trials in UAE, Bahrain, Egypt, and Jordan:** 72.8% against symptomatic disease ≥14 d after 2 doses, and 79% against hospitalization.	WHO EUL (Approval pending) China	[26,40,110,114,115]
Inactivated virus	4	Inactivated SARS-CoV-2 vaccine (Vero cell)	2 doses (50, 100, or 150 EU)	Day 0 + 14	IM	Institute of Medical Biology + Chinese Academy of Medical Sciences	Whole inactivated SARS-CoV-2 with Al(OH)_3_ adjuvant	Phase III (Enrolling by invitation)	NR	Not yet approved in any country	[26,116,117]
Inactivated virus	5	QazCovid-in	2 doses	Day 0 + 21	IM	Research Institute for Biological Safety Problems, Rep of Kazakhstan	Whole inactivated SARS-CoV-2	Phase III (Active, not recruiting)	**Efficacy from clinical trials in the Republic of Kazakhstan:** 96%	Republic of Kazakhstan	[26,118,119]
Inactivated virus	6	BBV152 (COVAXIN)	2 doses (3 or 6 μg)	Day 0 + 14	IM	Bharat Biotech International Limited	Whole inactivated SARS-CoV-2 with Algel-IMDG adjuvant	Phase III (Active, not recruiting)	**Efficacy from clinical trials:** 77.8% against symptomatic disease, 93.4% against severe disease, 63.6% against asymptomatic disease. **Efficacy/effectiveness against variants:** 65.2% against disease caused by Delta (B.617.2) variant.	WHO EUL (Approval pending) Approved in 9 countries ^3^	[26,110,120,121,122,123]
Inactivated virus	7	Inactivated SARS-CoV-2 vaccine (Vero cell)	2 doses	Day 0 + 28	IM	Shenzhen Kangtai Biological Products Co., Ltd.	Whole inactivated SARS-CoV-2	Phase III (Not yet recruiting)	NR	China	[26,124]
Inactivated virus	8	VLA2001	2 doses	Day 0 + 21	IM	Valneva, National Institute for Health Research, United Kingdom	Whole inactivated SARS-CoV-2 with high S-protein density, in combination with two adjuvants, alum and CpG 1018	Phase III (Not yet recruiting)	NR	Not yet approved in any country	[26,125]
Inactivated virus	9	ERUCOV-VAC (TURKOVAC)	2 doses (3 μg)	Day 0 + 28	IM	Erciyes University + Health Institutes of Turkey	Whole inactivated SARS-CoV-2	Phase III (Recruiting)	NR	Not yet approved in any country	[26,126]
Inactivated virus	10	COVID-19 inactivated vaccine	2 doses (5 μg)	Day 0 + 28	IM	Shifa Pharmed Industrial Co	Whole inactivated SARS-CoV-2	Phase II–III (Recruitment complete)	NR	Iran	[26,127]
Inactivated virus	11	FAKHRAVAC (MIVAC)	2 doses (10 µg)	Day 0 + 14	IM	Organization of Defensive Innovation and Research	Whole inactivated SARS-CoV-2	Phase II (Recruiting)	NR	Not yet approved in any country	[26,128]
Inactivated virus	12	Inactivated (NDV-based) chimeric vaccine	2 doses	Day 0 + 28	IM	The Government Pharmaceutical Organization (GPO) + PATH + Dynavax	Whole inactivated NDV chimera stably expressing membrane-anchored SARS-CoV-2 S protein +/− CpG 1018 adjuvant	Phase I–II (NR)	NR	Not yet approved in any country	[26,129]
Inactivated virus	13	KD-414	2 doses	Day 0 + 28	IM	KM Biologics Co., Ltd.	Whole inactivated SARS-CoV-2	Phase I–II (Not Recruiting)	NR	Not yet approved in any country	[26,130]
Inactivated virus	14	Koçak-19	2 doses (4 or 6 µg)	Day 0 + 21	IM	Kocak Farma, Turkey	Whole inactivated SARS-CoV-2 with adjuvant	Phase I (Recruiting)	NR	Not yet approved in any country	[26,131]
Inactivated virus	15	Adjuvanted inactivated vaccine	2 doses (10 µg-3M or 20 µg-6M)	Day 0 + 20	SC	The Scientific and Technological Research Council of Turkey (TÜBITAK)	Whole inactivated SARS-CoV-2 with CpG ODN adjuvant	Phase I (Recruiting)	NR	Not yet approved in any country	[26,132]
Inactivated virus	16	Live recombinant (rNDV) vector vaccine	2 doses	Day 0 + 21	IM or IN	Laboratorio Avi-Mex	Live recombinant NDV vector expressing SARS-CoV-2 S protein	Phase I (Recruiting)	NR	Not yet approved in any country	[26,133]
Live-attenuated virus	1	COVI-VAC	1–2 doses	Day 0 or Day 0 + 28	IN	Codagenix, Inc + Serum Institute of India	Whole SARS-CoV-2 with all viral proteins	Phase I (Active, not recruiting)	NR	Not yet approved in any country	[26,134]
Live-attenuated virus	2	MV-014-212	1 dose	Day 0	IN	Meissa Vaccines, Inc.	RSV expressing SARS-CoV-2 S protein	Phase I (Recruiting)	NR	Not yet approved in any country	[26,55,135]
Viral vector (non-replicating)	1	ChAdO x 1 AZD1222	2 doses (standard dose: 5 × 10^10^ viral particles, low dose: 2.2 × 10^10^ viral particles)	Day 0 + 28	IM	AstraZeneca + University of Oxford	Chimpanzee adenovirus-vectored vaccine (ChAdOx1) expressing S protein	Phase IV (Recruiting)	**Efficacy from clinical trials in UK, Brazil, and South Africa:** 66.7%–70.4% overall efficacy ≥14 d after 2 doses, 62.1% after 2 standard doses76.0% after single low dose within 20–90 d, 90.0% after one low dose and one standard dose. **Real-world effectiveness:** **England:** 60–75% after 1 dose. **Scotland:** 88% against hospitalization 28–34 d after 1 dose. **U.S:** 76% in adults, and 85% in elderly (≥65 y). **Efficacy/effectiveness against variants:** **UK:** 70.4% against Alpha (B.1.1.7) variant, 81.5% against non-B.1.1.7 lineages. **South Africa:** 10.4% against Beta (B.1.351) variant. **England:** 76.0% after 1 dose, 86.0% after 2 doses against Beta variant. 71.0% after 1 dose, 92.0% after 2 doses against Delta variant. **Canada:** 68% ≥ 14 d after dose 1 against symptomatic infection caused by Alpha variant. 48% ≥ 14 d after 1 dose against symptomatic infection caused by Beta or Gamma (P.1) variants. 67% ≥ 14 d after 1 dose against symptomatic infection caused by Delta variant.	WHO EUL Approved in 118 countries ^4^ and issued an Endorsed by ART CARPHA EU recommendation EMA approved	[67,72,73,74,93,110,136,137,138,139,140,141,142,143,144,145]
Viral vector (non-replicating)	2	Convidicea (Ad5-nCoV)	1 dose (5 × 10^10^ viral particles per dose)	Day 0	IM	CanSino Biological Inc. + Beijing Institute of Biotechnology	Recombinant replication-defective human type 5 adenovirus (Ad5) expressing S protein	Phase IV (Active, not recruiting)	**Efficacy from clinical trials in Pakistan, Russia, Argentina, Mexico, and Chile:** 68.8% and 65.7% against symptomatic disease ≥14 d and ≥28 d after vaccination, respectively. 95.5% and 91.0% against severe disease ≥14 d and ≥28 d after vaccination, respectively.	WHO EUL (Approval pending) Approved in 8 countries ^5^	[26,110,146,147,148,149,150,151]
Viral vector (non-replicating)	3	Ad26.COV2.S	1 dose (5 × 10^10^ viral particles per dose)	Day 0	IM	Janssen Pharmaceutical	Recombinant replication-incompetent adenovirus serotype 26 (Ad26) vector encoding full-length and stabilized S protein	Phase IV (NR)	**Efficacy from clinical trials in Argentina, Brazil, Chile, Colombia, Mexico, Peru, South Africa, and the U.S:** 66.3-76.3% and 65.5-83.5% against moderate to severe/critical disease ≥14 d and ≥28d after vaccination, respectively. **Real-world efficacy:** **U.S. and India:** 76.7% against infection ≥14 d after vaccination. **Efficacy/effectiveness against variants:** **South Africa** (95% predominant B.1.351 variant): 52.0–73.1% and 64.0–81.7% against moderate to severe/critical disease ≥14 d and ≥28 d after vaccination, respectively. **Brazil** (69% predominant P.2 lineages): 66.2–68.1% and 81.9–87.6% against moderate to severe/critical disease ≥14 d and ≥28 d after vaccination, respectively.	FDA EUA WHO EUL Approved in 55 countries ^6^ Endorsed by ART EMA approved	[26,69,71,110,145,152,153]
Viral vector (non-replicating)	4	Gam-COVID-Vac (Sputnik V)	2 doses (1 × 10^11^ viral particles per dose)	Day 0 + 21 (first: rAd26-S; second: rAd5-S)	IM	Gamaleya Research Institute + Health Ministry of the Russian Federation	Recombinant Ad26 and recombinant Ad5 encoding full-length S protein (rAd26-S and rAd5-S)	Phase III (Active, not recruiting)	**Efficacy from clinical trials:** 91.6% overall efficacy against symptomatic disease, 100% against moderate-severe disease, 73.1% after 1 dose, 91.1% after 2 doses. **Efficacy/effectiveness against variants:** 90% against Delta variant.	WHO EUL (Approval pending) Approved in 69 countries ^7^	[26,110,154,155,156,157]
Viral vector (non-replicating)	5	GRAd-COV2	1–2 doses (1 × 10^11^ viral particles per dose)	Day 0 + 21	IM	ReiThera + Leukocare + Univercells	Replication defective Simian Adenovirus (GRAd) encoding S protein	Phase II–III (Active, not recruiting)	NR	Not yet approved in any country	[26,158,159,160]
Viral vector (non-replicating)	6	LV-SMENP-DC	1 dose (5 × 10^6^ cells of LV-DC vaccine and 1 × 10^8^ antigen-specific CTLs)	Day 0	SC (LV-DC vaccine) and IV (antigen-specific CTLs)	Shenzhen Geno-Immune Medical Institute	Modified dendritic cells (DC) with lentivirus vectors (LV) expressing minigenes SMENP and immune-modulatory genes. Cytotoxic T-cells (CTLs) are activated by LV-DC, presenting specific viral antigens	Phase I–II (Recruiting)	NR	Not yet approved in any country	[26,161]
Viral vector (non-replicating)	7	hAd5-S-Fusion + N-ETSD vaccine	1 dose (5 × 10^10^ IU/ dose SC, 1 × 10^10^ IU/ dose SL)	Day 0	SC, oral, or SL	ImmunityBio, Inc. + NantKwest, Inc.	Human second-generation adenovirus 5 (hAd5) encoding S and N antigens	Phase I–II (Not yet recruiting)	NR	Not yet approved in any country	[26,162,163,164]
Viral vector (non-replicating)	8	AdCLD-CoV19	1 dose (2.5 × 10^10^, 5 × 10^10^, or 1 × 10^11^ virus particles per dose)	Day 0	IM	Cellid Co., Ltd.	Replication-defective human adenovirus type 5/35 vector expressing S protein	Phase I–II (Recruiting)	NR	Not yet approved in any country	[26,165]
Viral vector (non-replicating)	9	COVIVAC	2 doses (1 × 10^7^ IU, 5 × 10^7^ IU, or 1 × 10^8^ IU per dose)	Day 0 + 28	IM	Institute of Vaccines and Medical Biologicals, Vietnam	NDV expressing membrane-anchored pre-fusion-stabilized trimeric S protein +/− CpG 1018 adjuvant	Phase I–II (Recruiting)	NR	Not yet approved in any country	[26,166]
Viral vector (non-replicating)	10	MVA-SARS-2-ST	2 doses (1 × 10^7^ IU, or 1 × 10^8^ IU per dose)	Day 0 + 28	IM	Universitätsklinikum Hamburg-Eppendorf + German Center for Infection Research	MVA vector expressing stabilized S protein	Phase I–II (Not yet recruiting)	NR	Not yet approved in any country	[26,167]
Viral vector (non-replicating)	11	MVA-SARS-2-S	2 doses (1 × 10^7^ IU, or 1 × 10^8^ IU per dose)	Day 0 + 28	IM	University of Munich (Ludwig-Maximilians)	MVA vector expressing S protein	Phase I (Recruiting)	NR	Not yet approved in any country	[26,168]
Viral vector (non-replicating)	12	VXA-CoV2-1	1–2 doses (1 × 10^10^ IU, or 1 × 10^11^ IU per dose)	Day 0 or Day 0 + 28	Oral	Vaxart	Non-replicating adenovirus vector expressing viral antigens and dsRNA adjuvant	Phase I (Active, not recruiting)	NR	Not yet approved in any country	[26,169,170]
Viral vector (non-replicating)	13	AdCOVID,	1–2 doses	Day 0 + NR	IN	Altimmune, Inc.	Adenovirus expressing the RBD of S protein	Phase I (Recruiting)	NR	Not yet approved in any country	[26,171]
Viral vector (non-replicating)	14	COH04S1 (MVA-SARS-2-S)	2 doses (1 × 10^7^, 1 × 10^8^, or 2.5 × 10^8^ PFU per dose)	Day 0 + 28	IM	City of Hope Medical Center + National Cancer Institute	Synthetic MVA carrying small pieces of SARS-CoV-2 DNA (the chemical form of genes)	Phase I (Recruiting)	NR	Not yet approved in any country	[26,172]
Viral vector (non-replicating)	15	ChAdV68-S ChAdV68-S-TCE (Homologous and heterologous prime-boost schedule)	2–3 doses (5 × 10^10^ or 1 × 10^11^ viral particles of ChAdV68-S, 10 µg or 30 µg SEM)	Day 0 + 28, or Day 0 + 56, or Day 0 + 112, or Day 0 + 56 + 112	IM	Gritstone Oncology	Chimpanzee Adenovirus serotype 68 (ChAd) and self-amplifying mRNA (SAM) vectors expressing either S protein alone, or S protein with additional T-cell epitopes (TCE)	Phase I (Recruiting)	NR	Not yet approved in any country	[26,173]
Viral vector (non-replicating)	16	SC-Ad6-1	1–2 doses	Day 0 or Day 0 + 21	IM	Tetherex Pharmaceuticals Corporation	Adenovirus vector vaccine	Phase I (Not yet recruiting)	NR	Not yet approved in any country	[26,174]
Viral vector (non-replicating)	17	BBV154	1–2 doses (1 × 10^10^ viral particles per dose)	Day 0 or Day 0 + 28	IN	Bharat Biotech International Limited	S protein	Phase I (Active, not recruiting)	NR	Not yet approved in any country	[26,175]
Viral vector (replicating)	18	DelNS1-2019-nCoV-RBD-OPT1	2 doses (1 × 10^7^ EID50 and 1 × 10^7.7^ EID50)	Day 0 + 28	IN	University of Hong Kong, Xiamen University + Beijing Wantai Biological Pharmacy	Genetically engineered live attenuated influenza virus vector expressing the RBD of S protein	Phase II (Recruiting)	NR	Not yet approved in any country	[26,176,177]
Viral vector (replicating)	19	rVSV-SARS-CoV-2-S Vaccine	2 doses (1 × 10^5^, 1 × 10^6^, 1 × 10^7^, or 1 × 10^8^ PFU/mL)	Day 0 + 28	IM	Institute for Biological Research	cDNA vector encoding the sequence of the N, P, M, and L genes of the VSV genome, and SARS-CoV-2 S protein	Phase I–II (Recruiting)	NR	Not yet approved in any country	[26,178]
Viral vector (replicating)	20	AV-COVID-19	1 dose (0.1, 0.33, or 1.0 mg)	Day 0	IM	Aivita Biomedical, Inc. + National Institute of Health Research and Development + Ministry of Health Republic of Indonesia	Autologous dendritic cells loaded with antigens from SARS-CoV-2 +/− GM-CSF	Phase I–II (Not yet recruiting)	NR	Not yet approved in any country	[26,179]
Viral vector (replicating)	21	Covid-19/aAPC vaccine	3 doses	Day 0 + 14 + 28	SC	Shenzhen Geno-Immune Medical Institute	Lentivirus vector system expressing viral minigenes to the artificial antigen-presenting cells (aAPCs)	Phase I (Recruiting)	NR	Not yet approved in any country	[26,180]
DNA based vaccine	1	nCov vaccine (ZyCoV-D)	3 doses (1 or 2 mg)	Day 0 + 28 + 56	ID	Zydus Cadila	S protein	Phase III (Not recruiting)	**Efficacy from clinical trials in India**: 66.6%	Not yet approved in any country	[26,81,181,182]
DNA based vaccine	2	INO-4800+ electroporation	2 doses (1 mg)	Day 0 + 28	ID	Inovio Pharmaceuticals + International Vaccine Institute + Advaccine Biopharmaceutical Co., Ltd.	S1 and S2 subunits of SARS-CoV-2 S protein	Phase II–III (Active, not recruiting)	NR	Not yet approved in any country	[26,183,184]
DNA based vaccine	3	AG0301-COVID19	2 doses (2 mg)	Day 0 + 14	IM	AnGes + Takara Bio + Osaka University	S protein	Phase II–III (Active, not recruiting)	NR	Not yet approved in any country	[26,185]
DNA based vaccine	4	GX-19	2 doses	Day 0 + 28	IM	Genexine Consortium	S protein	Phase I–II (Recruiting)	NR	Not yet approved in any country	[26,186]
DNA based vaccine	5	Covigenix VAX-001	2 doses	Day 0 + 14	IM	Entos Pharmaceuticals Inc.	Full-length S protein	Phase I–II (Recruiting)	NR	Not yet approved in any country	[26,187]
DNA based vaccine	6	GLS-5310	2 doses (0.6 or 1.2 mg)	Day 0 + 56 or Day 0 + 84	ID	GeneOne Life Science, Inc.	S protein and a second antigenic target of SARS-CoV-2	Phase I–II (Recruiting)	NR	Not yet approved in any country	[26,188,189]
DNA based vaccine	7	COVID-eVax	2 doses (0.5, 1, or 2 mg)	Day 0 + 28	IM	Takis + Rottapharm Biotech	RBD of S protein	Phase I–II (Recruiting)	NR	Not yet approved in any country	[26,190]
DNA based vaccine	8	CORVax	2 doses	Day 0 + 14	ID	Providence Health and Services	S protein +/− the combination of electroporated IL-12p70 plasmid	Phase I (Active, not recruiting)	NR	Not yet approved in any country	[26,191]
DNA based vaccine	9	bacTRL	1–2 doses	Day 0 or Day 0 + 28	Oral	Symvivo Corporation	S protein	Phase I (Active, not recruiting)	NR	Not yet approved in any country	[26,192]
DNA based vaccine	10	COVIGEN (COVALIA)	2 doses (0.8, 2, or 4 mg)	Day 0 + 28	IM or ID	University of Sydney, Bionet Co., Ltd.	S protein	Phase I (Not yet recruiting)	NR	Not yet approved in any country	[26,193]
RNA vaccine	1	mRNA-1273	2 doses (100 μg)	Day 0 + 28	IM	Moderna + National Institute of Allergy and Infectious Diseases (NIAID)	Full-length S protein with proline substitutions	Phase IV (Recruiting)	**Efficacy from clinical trials in the U.S.:** 92.1% against symptomatic disease ≥14 d after 1 dose, 94.1% ≥ 14 d after 2 doses, and 100% against severe disease. **Real-world efficacy:** **U.S.:** 80% ≥ 14 d after 1 dose and 90% ≥ 14 d after 2 doses. 83% ≥ 14 d after 1 dose and 82% after 2 doses. 88.7% against infection ≥ 36 d after 1 dose. **Canada:** 72% against infection after 1 dose and 94% after 2 doses. **Efficacy/ effectiveness against variants:** **Qatar:** 88.1% ≥ 14 d after 1 dose, 100% after 2 doses against Alpha variant. 61.3% ≥ 14 d after 1 dose, 96.4% after 2 doses against Betavariant. **Canada:** 83% ≥ 14 d after 1 dose and 92% ≥ 7 d after 2 doses against symptomatic infection caused by Alpha variant. 77% ≥ 14 d after 1 dose against symptomatic infection caused by Beta or Gammavariants. 72% ≥ 14 d after 1 dose against symptomatic infection caused by Delta variant.	FDA EUAWHO EUL Approved in 57 countries ^9^EMA approved	[26,73,88,92,94,110,137,145,194,195,196,197,198]
RNA vaccine	2	BNT162b2 (3 LNP-mRNAs), also known as “Comirnaty”	2 doses (30 μg)	Day 0 + 21	IM	Pfizer/BioNTech + Fosun Pharma	Full-length S protein with proline substitutions	Phase IV (Recruiting)	**Efficacy from clinical trials:** 52.4% after 1 dose and 94.6% ≥ 7 d after 2 doses in adults. **Real-world efficacy:** **England**: 60–70% against infection after 1 dose, 85–90% after 2 doses in elderly (≥80 y). 72% against infection ≥21 d after 1 dose, and 86% ≥ 7 d after 2 doses. 91% against infection 15–28 d after 1 dose. **UK**: 70% ≥ 21 d after 1 dose, 85% ≥ 7 d after 2 doses. **Denmark**: 17% ≥ 14 d after 1 dose, 64–90% ≥ 7 d after 2 doses. **Scotland**: 91% against hospitalization 28–34 d after 1 dose. **U.S.:** 80% ≥ 14 d after 1 dose, 93% ≥ 14 d after 2 doses. 88.7% against infection ≥ 36 d after 1 dose. **Sweden**: 42% against infection ≥ 14 d after 1 dose, 86% ≥ 7 d after 2 doses. **Canada**: 59% ≥ 14 d after 1 dose and 91% after 2 doses. **Qatar**: 39.4% against disease after 1 dose and 97.4% ≥ 14 d after 2 doses. **Efficacy/ effectiveness against variants:** **England**: 83.0% against hospitalization after 1 dose, 95.0% after 2 doses against Alpha variant. 94.0% against hospitalization after 1 dose, 96.0% after 2 doses against Deltavariant. **Canada**: 89% ≥ 7 d after 2 doses against symptomatic infection caused by Alpha variant. 60% ≥ 14 d after 1 dose and 84% ≥ 7 d against symptomatic infection caused by Beta or Gammavariants. 56% ≥ 14 d after 1 dose and 87% ≥ 7 d against symptomatic infection caused by Delta variant. **Qatar**: 29.5% after 1 dose and 89.5% ≥ 14 d after 2 doses against infection caused by Alpha variant. 16.9% after 1 dose and 75.0% after 2 doses against infection caused by Beta variant.	FDA EUA WHO EUL Approved in 93 countries ^10^ CARPHA EU recommendation EMA approved	[26,73,89,92,93,110,141,142,145,196,197,199,200,201,202,203,204,205,206,207,208,209]
RNA vaccine	3	CVnCoV (CureVac)	2 doses (12 μg)	Day 0 + 28	IM	CureVac AG	LNP-encapsulated mRNA vaccine encoding the full-length, pre-fusion stabilized S protein	Phase III (Active, not recruiting)	**Efficacy from clinical trials conducted in 10 countries in Latin America and Europe:** 47% against symptomatic disease across all age groups and 15 variants, 53% against any disease severity, 77% against moderate and severe disease.	WHO EUL (Pending approval) Not yet approved in any country	[26,110,210,211,212]
RNA vaccine	4	ARCoV or ARCoVax	1 dose (15 μg)	Day 0	IM	Academy of Military Science (AMS), Walvax Biotechnology and Suzhou Abogen Biosciences	LNP-encapsulated mRNA vaccine encoding the RBD of S protein	Phase III (Not yet recruiting)	NR	Not yet approved in any country	[26,213,214]
RNA vaccine	5	mRNA-1273.211	1 dose (50 μg)	Day 0	IM	ModernaTX, Inc.	A multivalent booster candidate combining mRNA-1273 + mRNA-1273.351	Phase II-III (Active, not recruiting)	NR	Not yet approved in any country	[26,215]
RNA vaccine	6	mRNA-1273.351	1–2 doses (20 or 50 μg)	Day 0, or Day 0 + 28, or Day 56 after 2nd dose of mRNA-1273	IM	Moderna + NIAID	Full-length prefusion stabilized S protein of SARS-CoV-2 B.1.351 variant	Phase II (Active, not recruiting)	NR	Not yet approved in any country	[26,216,217,218]
RNA vaccine	7	ARCT-021	1–2 doses ± booster dose (5 or 7.5 μg)	Day 0, or Day 0 + 28, or Day 0 + 28 ± 208 (booster)	IM	Arcturus Therapeutics	S protein	Phase II (Two trials: one is recruiting, and the other is active, not recruiting)	NR	Not yet approved in any country	[26,219,220,221]
RNA vaccine	8	MRT5500	2 doses (15, 45, or 135 µg)	Day 0 + 21	IM	Sanofi Pasteur and Translate Bio	S protein	Phase I–II (Recruiting)	NR	Not yet approved in any country	[26,222,223,224]
RNA vaccine	9	DS-5670a	2 doses (10, 30, 60 or 100 µg)	Day 0 + 21	IM	Daiichi Sankyo Co., Ltd.	NR	Phase I–II (Active, not recruiting)	NR	Not yet approved in any country	[26,225,226]
RNA vaccine	10	EXG-5003	1 dose	Day 0	ID	Elixirgen Therapeutics, Inc	Temperature-sensitive ssRNA vaccine expressing the RBD of S protein	Phase I–II (Recruiting)	NR	Not yet approved in any country	[26,227]
RNA vaccine	11	LNP-nCoVsaRNA (COVAC1)	2 doses (0.1–10.0 µg)	ND	IM	Imperial College London	S protein	Phase I (No longer recruiting)	NR	Not yet approved in any country	[26,228,229]
RNA vaccine	12	ChulaCov19 mRNA vaccine	2 doses (10, 25, 50, or 100 µg)	Day 0 + 21	IM	Chulalongkorn University	S protein	Phase I (Not yet recruiting)	NR	Not yet approved in any country	[26,230,231]
RNA vaccine	13	PTX-COVID19-B	2 doses (16, 40, or 100 μg)	Day 0 + 28	IM	Providence Therapeutics	Full-length membrane-anchored S protein	Phase I (Active, not recruiting)	NR	Not yet approved in any country	[26,232,233]
RNA vaccine	14	CoV2 SAM (LNP)	2 doses (1.0 μg)	Day 0 + 30	IM	GSK	S protein	Phase I (Active, not recruiting)	NR	Not yet approved in any country	[26,234]
RNA vaccine	15	HDT-301	2 doses (1, 5, or 25 μg)	Day 0 + 28	IM	SENAI CIMATEC	Full-length S protein	Phase I (Not yet recruiting)	NR	Not yet approved in any country	[26,235]
RNA vaccine	16	mRNA-1283	1–2 doses (10, 30, or 100 μg)	Day 0 or Day 0 + 28	IM	ModernaTX, Inc.	RBD and NTD of S protein	Phase I (Recruiting)	NR	Not yet approved in any country	[26,236,237]
RNA vaccine	17	SW-0123	2 doses	NR	IM	Shanghai East Hospital + Stemirna Therapeutics	NR	Phase I (Recruiting)	NR	Not yet approved in any country	[26,238,239]
RNA vaccine	18	LNP-nCOV saRNA-02 (COVAC-Uganda)	2 doses (5.0 µg)	Day 0 + 28	IM	MRC/UVRI and LSHTM Uganda Research Unit	S protein	Phase I (Not yet recruiting)	NR	Not yet approved in any country	[26,240]
Protein subunit	1	NVX-CoV2373	2 doses (5 µg)	Day 0 + 21	IM	Novavax	S protein with Matrix-M adjuvant	Phase III (Recruiting)	**Efficacy from clinical trials:** **UK**: 89.7% against symptomatic disease ≥7 d after 2 doses. **Real-world efficacy:** **U.S.:** 100% against mild and severe disease. **Efficacy/effectiveness against variants:** **UK:** 86.2% against Alpha variant, 96.4% against non-B.1.1.7 variants. **South Africa:** 51.0% against Beta variant after 2 doses. 85.6% against symptomatic disease caused by Alpha variant. 60% against any disease severity in predominantly circulating Beta variant. **U.S.:** 93% against Alpha, Beta, and other VOCs/ VOIs.	WHO EUL (Approval pending) Not yet approved in any country	[26,102,103,110,241,242,243]
Protein subunit	2	ZF2001 (Recombinant SARS-CoV-2 vaccine)	3 doses (25 µg)	Day 0 + 30 + 93	IM	Anhui Zhifei Longcom Biopharmaceutical + Institute of Microbiology, Chinese Academy of Sciences	RBD-Dimer with alum adjuvant	Phase III (Recruiting)	NR	China (EUA), Uzbekistan	[26,244,245]
Protein subunit	3	VAT00008	2 doses	Day 0 + 21	IM	Sanofi Pasteur + GSK	Monovalent and bivalent S protein with adjuvant	Phase III (Not yet recruiting)	NR	Not yet approved in any country	[26,246,247]
Protein subunit	4	FINLAY-FR-2	2 doses (25 μg) + booster dose (FINLAY-FR-1A, 50 μg))	Day 0 + 28 Day 56 (booster dose)	IM	Instituto Finlay de Vacunas	FINLAY-FR-2: chemically conjugated RBD to tetanus toxoid plus adjuvant FINLAY-FR-1A: dimeric RBD + alum adjuvant	Phase III (Pending)	62%	Not yet approved in any country	[26,248,249,250]
Protein subunit	5	Recombinant SARS-CoV-2 vaccine (Sf9 Cell)	3 doses	Day 0 + 28 + 42	IM	West China Hospital + Sichuan University	RBD with alum adjuvant	Phase III (Enrolling by invitation)	NR	Not yet approved in any country	[26,251]
Protein subunit	6	EpiVacCorona	2 doses	Day 0 + 21	IM	Federal Budgetary Research Institution State Research Center of Virology and Biotechnology	Peptide antigens of SARS-CoV-2 proteins with alum adjuvant	Phase III (Active, not recruiting)	**Efficacy from clinical trials:** 100%	Russia, Turkmenistan	[26,252,253]
Protein subunit	7	CIGB-66	3 doses (50 µg RBD + 0.3 mg aluminum hydroxide)	Day 0 + 14 + 28 or Day 0 + 28 + 56	IM	Center for Genetic Engineering and Biotechnology (CIGB)	RBD with aluminum hydroxide adjuvant	Phase III (Pending)	**Efficacy from clinical trials:** 91.6%	Not yet approved in any country	[26,254,255]
Protein subunit	8	NanoCovax	2 doses (25 µg)	Day 0 + 28	IM	Nanogen Pharmaceutical Biotechnology	Recombinant S protein with alum adjuvant	Phase III (Recruiting)	NR	Not yet approved in any country	[26,256]
Protein subunit	9	SCB-2019	2 doses (30 μg)	Day 0 + 21	IM	Clover Biopharmaceuticals Inc. + GSK + Dynavax	Trimeric S protein with CpG 1018 and Alum adjuvants	Phase II–III (Not yet recruiting)	NR	Not yet approved in any country	[26,257,258,259]
Protein subunit	10	UB-612	2 doses (100 µg)	Day 0 + 28	IM	Vaxxinity, Inc. + Diagnósticos da América S/A (DASA)	RBD of S protein	Phase II–III (Not yet recruiting)	NR	Not yet approved in any country	[26,260]
Protein subunit	11	FINLAY-FR-1	2 doses (10 or 20 μg)	Day 0 + 28	IM	Instituto Finlay de Vacunas	RBD with adjuvant	Phase II (Pending)	NR	Not yet approved in any country	[26,261]
Protein subunit	12	COVAX-19	2 doses (25 μg)	Day 0 + 21	IM	Vaxine Pty Ltd. + CinnaGen Co.	Recombinant S protein with Advax-CpG adjuvant	Phase II (Recruiting)	NR	Not yet approved in any country	[26,262]
Protein subunit	13	MVC-COV1901	2 doses (5, 15, or 25 μg)	Day 0 + 28	IM	Medigen Vaccine Biologics + Dynavax + NIAID	Recombinant S protein with CpG 1018 and alum adjuvants	Phase II (Active, not recruiting for adults, recruiting for elderly)	NR	Not yet approved in any country	[26,263,264,265]
Protein subunit	14	Razi Cov Pars	3 doses	Day 0 + 21 (IM) + 51 (IN)	IM and IN	Razi Vaccine and Serum Research Institute	Recombinant S protein	Phase II (Complete)	NR	Not yet approved in any country	[26,266]
Protein subunit	15	V-01	2 doses (10 or 25 μg)	Day 0 + 21	IM	Guangdong Provincial Center for Disease Control and Prevention/ Gaozhou Center for Disease Control and Prevention	Recombinant S protein	Phase II (Not yet recruiting)	NR	Not yet approved in any country	[26,267]
Protein subunit	16	CIGB-669	3 doses (50 µg RBD + 40 µg AgnHB)	Day 0 + 14 + 28 or Day 0 + 28 + 56	IN	Center for Genetic Engineering and Biotechnology (CIGB)	Recombinant RBD with AgnHB	Phase I–II (Pending)	NR	Not yet approved in any country	[26,268]
Protein subunit	17	KBP-COVID-19	2 doses (15 μg in phase I, 45 μg in phase II)	Day 0 + 21	IM	Kentucky Bioprocessing Inc.	RBD of S protein	Phase I–II (Recruiting)	NR	Not yet approved in any country	[26,269,270]
Protein subunit	18	BECOV2	2 doses	Day 0 + 28	IM	Biological E. Limited	Recombinant RBD	Phase I–II (Closed)	NR	Not yet approved in any country	[26,271]
Protein subunit	19	S-268019	2 doses	Day 0 + 21	IM	Shionogi	Recombinant S protein	Phase I–II (Recruiting)	NR	Not yet approved in any country	[26,272]
Protein subunit	20	AKS-452	1–2 doses (22.5, 45, or 90 µg)	NR	SC or IM	University Medical Center Groningen + Akston Biosciences Inc.	RBD-Fc fusion protein	Phase I–II (Enrolling by invitation)	NR	Not yet approved in any country	[26,273]
Protein subunit	21	COVAC-1 and COVAC-2	2 doses (25, 50, or 100 µg)	Day 0 + 28	IM	University of Saskatchewan	S1 protein with SWE adjuvant	Phase I–II (Recruiting)	NR	Not yet approved in any country	[26,274]
Protein subunit	22	GBP510	2 doses (10, or 25 µg)	Day 0 + 28	IM	SK Bioscience Co., Ltd. And CEPI	Recombinant RBD with AS03 aluminum hydroxide adjuvant	Phase I–II (Recruiting)	NR	Not yet approved in any country	[26,275]
Protein subunit	23	QazCoVac-P	1–2 doses	Day 0 + 21	IM	Research Institute for Biological Safety Problems		Phase I–II (Active, not recruiting)	NR	Not yet approved in any country	[26,276]
Protein subunit	24	EuCorVac-19	2 doses	Day 0 + 21	IM	POP Biotechnologies and EuBiologics Co., Ltd	Recombinant S protein with an adjuvant	Phase I–II (Recruiting)	NR	Not yet approved in any country	[26,277]
Protein subunit	25	Recombinant SARS-CoV-2 Vaccine (CHO cell)	3 doses	Day 0 + 30 + 60	IM	National Vaccine and Serum Institute, China	Recombinant SARS-CoV-2	Phase I–II (Recruiting)	NR	Not yet approved in any country	[26,278]
Protein subunit	26	SARS-CoV-2 Sclamp vaccine	2 doses (5, 15, or 45 μg)	Day 0 + 28	IM	University of Queensland + Syneos Health + CEPI	Recombinant S protein with MF59 adjuvant	Phase I (Recruiting)	NR	Not yet approved in any country	[26,279,280,281]
Protein subunit	27	IMP CoVac-1	1 dose (500 µL)	Day 0	SC	University Hospital Tuebingen	SARS-CoV-2 HLA-DR peptides	Phase I (Recruiting)	NR	Not yet approved in any country	[26,282]
Protein subunit	28	AdimrSC-2f	NR	NR	NR	Adimmune Corporation	Recombinant RBD with alum adjuvant	Phase I (Recruiting)	NR	Not yet approved in any country	[26,283]
Protein subunit	29	NBP2001	2 doses (30 or 50 μg)	Day 0 + 28	IM	SK Bioscience Co., Ltd.	Recombinant RBD protein with alum adjuvant	Phase I (Active, not recruiting)	NR	Not yet approved in any country	[26,284]
Protein subunit	30	ReCOV	2 doses (20 or 40 μg)	Day 0 + 21	IM	Jiangsu Rec-Biotechnology	Recombinant two-component S and RBD protein	Phase I (Not yet recruiting)	NR	Not yet approved in any country	[26,285]
Protein subunit	31	Spike-Ferritin-Nanoparticle (SpFN)	2–3 doses (25 or 50 μg)	Day 0 + 28 + 180	IM	Walter Reed Army Institute of Research (WRAIR)	S proteins with a liposomal formulation QS21 (ALFQ) adjuvant	Phase I (Recruiting)	NR	Not yet approved in any country	[26,286,287,288]
Protein subunit	32	CoVepiT	1–2 doses	Day 0 or Day 0 + 21	SC	OSE Immunotherapeutics	Target 11 viral protein (S, M, N, and several non-structural proteins)	Phase I (Recruiting)	NR	Not yet approved in any country	[26,289]
Protein subunit	33	CoV2-OGEN1	1–2 doses (50, 100, or 200 μg)	Day 0 or Day 0 + 14	Oral	VaxForm	Recombinant RBD protein	Phase I (Not yet recruiting)	NR	Not yet approved in any country	[26,290]
Virus-like particle	1	CoVLP	2 doses (3.75 µg)	Day 0 + 21	IM	Medicago Inc.	Trimeric S protein with AS03 adjuvant	Phase II–III (Recruiting)	NR	Not yet approved in any country	[26,291,292]
Virus-like particle	2	RBD SARS-CoV-2 HBsAg VLP	2 doses (5 or 25 µg)	Day 0 + 28	IM	Serum Institute of India + Accelagen Pty + SpyBiotech	RBD conjugated to the hepatitis B surface antigen	Phase I–II (Recruiting)	NR	Not yet approved in any country	[26,293]
Virus-like particle	3	VBI-2902a	2 doses (5 or 10 μg)	Day 0 + 28	IM	VBI Vaccines Inc.	Enveloped S glycoprotein with aluminum phosphate adjuvant	Phase I–II (Active, not recruiting)	NR	Not yet approved in any country	[26,294]
Virus-like particle	4	SARS-CoV-2 VLP Vaccine	2 doses	NR	SC	The Scientific and Technological Research Council of Turkey	SARS-CoV-2 VLP adjuvanted with alum and CpG ODN-K3	Phase I (Recruiting)	NR	Not yet approved in any country	[26,295]
Virus-like particle	5	ABNCoV2	2 doses	Day 0 + 28	IM	Radboud University	capsid virus-like particle (cVLP) +/− adjuvant MF59	Phase I (Recruiting)	NR	Not yet approved in any country	[26,296]

**Abbreviations**: IM: Intramuscular, IN: Intranasal, IV: Intravascular, SC: Subcutaneous, ID: Intradermal, SL: Sublingual, NR: Not reported, d: days, FDA: Food and Drug Administration, WHO: World Health Organization, EUA: Emergency Use Authorization, EUL: Emergency Use Listing, ART: Africa Regulatory Taskforce, CRS: Caribbean Regulatory System, EMA: European Medicines Agency, EU: Equivalent units, IU: Infectious unit, PFU: Plaque-forming unit, S: Spike, RBD: Receptor-binding domain, N: nucleocapsid, M: membrane, NTD: N-terminal domain, Al(OH)_3_: aluminum hydroxide, Algel-IMDG: chemosorbed imidazoquinoline onto aluminum hydroxide gel, CpG 1018: cytosine phosphoguanine 1018, CpG ODN: CpG oligodeoxynucleotide, NVD: Newcastle Disease Virus, RSV: Respiratory syncytial virus, MVA: Modified vaccinia virus Ankara, VSV: Vesicular stomatitis virus, GM-CSF: Granulocyte-macrophage colony-stimulating factor, ssRNA: Self-amplifying ribonucleic acid, LNP: Lipid nanoparticles, AgnHBL antigen of Hepatitis B, VOCs: variants of concern, VOIs: variants of interest.

* Efficacy against COVID-19 varies by age and time after vaccinations.^1^ Albania, Armenia, Azerbaijan, Bangladesh, Benin, Brazil, Cambodia, Chile, China, Colombia, Dominican Republic, Ecuador, Egypt, El Salvador, Georgia, Hong Kong, Indonesia, Kazakhstan, Lao People’s Democratic Republic, Malaysia, Mexico, Nepal, Oman, Pakistan, Panama, Paraguay, Philippines, South Africa, Tajikistan, Thailand, Timor-Leste, Togo, Tunisia, Turkey, Ukraine, Uruguay, and Zimbabwe. ^2^ Angola, Argentina, Bahrain, Bangladesh, Belarus, Belize, Bolivia, Brazil, Brunei Darussalam, Cambodia, Cameroon, China, Comoros, Egypt, Equatorial Guinea, Gabon, Gambia, Georgia, Guyana, Hungary, Indonesia, Iran, Iraq, Jordan, Kyrgyzstan, Lao People’s Democratic Republic, Lebanon, Maldives, Mauritania, Mauritius, Mongolia, Montenegro, Morocco, Mozambique, Namibia, Nepal, Niger, North Macedonia, Pakistan, Paraguay, Peru, Philippines, Republic of the Congo, Senegal, Serbia, Seychelles, Sierra Leone, Solomon Islands, Somalia, Sri Lanka, Thailand, Trinidad and Tobago, United Arab Emirates, Venezuela (Bolivarian Republic of Venezuela), Vietnam, and Zimbabwe. ^3^ Guyana, India, Iran, Mauritius, Mexico, Nepal, Paraguay, Philippines, and Zimbabwe. ^4^ Albania, Angola, Argentina, Armenia, Australia, Austria, Azerbaijan, Belgium, Belize, Benin, Bermuda, Bosnia and Herzegovina, Botswana, Brazil, Brunei Darussalam, Bulgaria, Burkina Faso, Cambodia, Canada, Central African Republic, Chile, Colombia, Costa Rica, Croatia, Cyprus, Czechia, Côte d’Ivoire, Democratic Republic of the Congo, Dominican Republic, Ecuador, Egypt, El Salvador, Estonia, Eswatini, Fiji, Finland, France, Gambia, Georgia, Germany, Ghana, Greece, Grenada, Guatemala, Guinea-Bissau, Guyana, Haiti, Hungary, Iceland, India, Indonesia, Iran, Iraq, Ireland, Italy, Jamaica, Japan, Jordan, Kenya, Kosovo, Kuwait, Latvia, Lesotho, Libya, Liechtenstein, Lithuania, Luxembourg, Malawi, Malaysia, Mali, Malta, Mauritius, Mexico, Mongolia, Morocco, Nauru, Netherlands, Niger, Nigeria, North Macedonia, Oman, Pakistan, Panama, Papua New Guinea, Paraguay, Peru, Philippines, Poland, Portugal, Republic of Korea, Republic of Moldova, Romania, Rwanda, Sao Tome and Principe, Saudi Arabia, Senegal, Serbia, Sierra Leone, Slovakia, Slovenia, South Sudan, Spain, Sudan, Sweden, Taiwan, Tajikistan, Thailand, Timor-Leste, Togo, Tunisia, Uganda, United Arab Emirates, United Kingdom of Great Britain and Northern Ireland, Uzbekistan, Vanuatu, Viet Nam, Yemen, and Zambia. ^5^ Argentina, Chile, China, Ecuador, Hungary, Malaysia, Mexico, and Pakistan. ^6^ Austria, Bahrain, Bangladesh, Belgium, Brazil, Bulgaria, Canada, Chile, Colombia, Croatia, Cyprus, Czechia, Denmark, Estonia, Faroe Islands, Finland, France, Germany, Greece, Hungary, Iceland, Ireland, Italy, Kuwait, Latvia, Libya, Liechtenstein, Lithuania, Luxembourg, Malaysia, Maldives, Malta, Mexico, Netherlands, New Zealand, Nigeria, Norway, Philippines, Poland, Portugal, Republic of Korea, Romania, Saint Vincent and the Grenadines, Slovakia, Slovenia, South Africa, Spain, Sweden, Switzerland, Thailand, Tunisia, Ukraine, United Kingdom of Great Britain and Northern Ireland, United States of America, and Zambia. ^7^ Albania, Algeria, Angola, Antigua and Barbuda, Argentina, Armenia, Azerbaijan, Bahrain, Bangladesh, Belarus, Bolivia, Brazil, Cameroon, Djibouti, Ecuador, Egypt, Gabon, Ghana, Guatemala, Guinea, Guyana, Honduras, Hungary, India, Iran, Iraq, Jordan, Kazakhstan, Kenya, Kyrgyzstan, Lao People’s Democratic Republic, Lebanon, Libya, Maldives, Mali, Mauritius, Mexico, Mongolia, Montenegro, Morocco, Myanmar, Namibia, Nepal, Nicaragua, North Macedonia, Oman, Pakistan, Panama, Paraguay, Philippines, Republic of Moldova, Republic of the Congo, Russian Federation, Saint Vincent and the Grenadines, San Marino, Serbia, Seychelles, Slovakia, Sri Lanka, Syrian Arab Republic, Tunisia, Turkey, Turkmenistan, United Arab Emirates, Uzbekistan, Venezuela, Vietnam, West Bank, and Zimbabwe. ^8^ Afghanistan, Antigua and Barbuda, Argentina, Bahrain, Bangladesh, Barbados, Bhutan, Bolivia, Botswana, Brazil, Cabo Verde, Canada, Côte d’Ivoire, Dominica, Egypt, Ethiopia, Ghana, Grenada, Honduras, Hungary, India, Jamaica, Lebanon, Maldives, Morocco, Myanmar, Namibia, Nepal, Nicaragua, Nigeria, Saint Kitts and Nevis, Saint Lucia, Saint Vincent and the Grenadines, Seychelles, Solomon Islands, Somalia, South Africa, Sri Lanka, Suriname, The Bahamas, Togo, Tonga, Trinidad and Tobago, Ukraine. ^9^ Austria, Bangladesh, Belgium, Botswana, Bulgaria, Canada, Croatia, Cyprus, Czechia, Denmark, Estonia, Faroe Islands, Finland, France, Germany, Greece, Greenland, Guatemala, Honduras, Hungary, Iceland, India, Ireland, Italy, Kuwait, Latvia, Libya, Liechtenstein, Lithuania, Luxembourg, Maldives, Mongolia, Netherlands, Norway, Philippines, Poland, Portugal, Qatar, Republic of Korea, Romania, Rwanda, Saint Vincent and the Grenadines, Seychelles, Singapore, Slovakia, Slovenia, Spain, Sweden, Switzerland, Taiwan, Thailand, United Arab Emirates, United Kingdom of Great Britain and Northern Ireland, United States of America, Viet Nam, and West Bank. ^10^ Albania, Argentina, Australia, Austria, Azerbaijan, Bahrain, Bangladesh, Belgium, Bermuda, Bosnia and Herzegovina, Botswana, Brazil, Brunei Darussalam, Bulgaria, Cabo Verde, Canada, Chile, Colombia, Costa Rica, Croatia, Cyprus, Czechia, Denmark, Dominican Republic, Ecuador, El Salvador, Estonia, Faroe Islands, Finland, France, Georgia, Germany, Greece, Greenland, Hong Kong, Hungary, Iceland, Iraq, Ireland, Italy, Japan, Jordan, Kuwait, Latvia, Lebanon, Libya, Liechtenstein, Lithuania, Luxembourg, Malaysia, Maldives, Malta, Mexico, Monaco, Mongolia, Netherlands, New Zealand, North Macedonia, Norway, Oman, Pakistan, Panama, Paraguay, Peru, Philippines, Poland, Portugal, Qatar, Republic of Korea, Republic of Moldova, Romania, Rwanda, Saint Vincent and the Grenadines, Saudi Arabia, Serbia, Singapore, Slovakia, Slovenia, South Africa, Spain, Sri Lanka, Sweden, Switzerland, Tunisia, Turkey, Ukraine, United Arab Emirates, United Kingdom of Great Britain and Northern Ireland, United States of America, Uruguay, Vatican, Viet Nam, and West Bank.

## 3. Conclusions

With the ongoing SARS-CoV-2 pandemic, safe and effective vaccines could be the major aid in retrenching this outbreak and probably the best bet to return us to ‘normal life’. The impulse of an accelerated vaccine development process, though needed, is faced with a broad spectrum of challenges that necessitates collective strives from both the public and the private sectors to fully understand the potential utility of these vaccines not only for overcoming the current pandemic but also for preventing future waves.

## Data Availability

Data used in the review were retrieved from the World Health Organization (WHO) vaccine tracker and landscape website (https://www.who.int/publications/m/item/draft-landscape-of-covid-19-candidate-vaccines) (accessed on 15 June 2021) and/or other publicly available resources as detailed through in-text citation and the references section of the review. Figures were created with BioRender.com.
